# INTERCEPT H3: a multicenter phase I peptide vaccine trial for the treatment of H3-mutated diffuse midline gliomas

**DOI:** 10.1186/s42466-023-00282-4

**Published:** 2023-10-19

**Authors:** Niklas Grassl, Katharina Sahm, Heike Süße, Isabel Poschke, Lukas Bunse, Theresa Bunse, Tamara Boschert, Iris Mildenberger, Anne-Kathleen Rupp, Max Philipp Ewinger, Lisa-Marie Lanz, Monika Denk, Ghazaleh Tabatabai, Michael W. Ronellenfitsch, Ulrich Herrlinger, Martin Glas, Dietmar Krex, Peter Vajkoczy, Antje Wick, Inga Harting, Felix Sahm, Andreas von Deimling, Martin Bendszus, Wolfgang Wick, Michael Platten

**Affiliations:** 1 Clinical Cooperation Unit Neuroimmunology and Brain Tumor Immunology, DKTK, DKFZ, Heidelberg, Germany; 2 Department of Neurology, Medical Faculty Mannheim, MCTN, Heidelberg University, Mannheim, Germany; 3 DKFZ-Hector Cancer Institute at University Medical Center Mannheim, Mannheim, Germany; 4 National Center for Tumor Diseases (NCT) Trial Center, NCT, Heidelberg, Germany; 5 Immune Monitoring Unit, German Cancer Research Center (DKFZ) and National Center for Tumor Diseases (NCT), Heidelberg, Germany; 6 Helmholtz Institute for Translational Oncology (HI-TRON), Mainz, Germany; 7 Faculty of Biosciences, Heidelberg University, Heidelberg, Germany; 8 Department of Peptide-based Immunotherapy, University and University Hospital Tübingen, Tübingen, Germany; 9 Institute for Cell Biology, Department of Immunology, University of Tübingen, Tübingen, Germany; 10 Partner site Tübingen, German Cancer Consortium (DKTK) and German Cancer Research Center (DKFZ), Tübingen, Germany; 11 Department of Neurology & Neuro-Oncology, University Hospital Tübingen, Tübingen, Germany; 12 Center for Neuro-Oncology, Comprehensive Cancer Center, University of Tübingen, Tübingen, Germany; 13 Dr. Senckenberg Institute for Neurooncology, University Hospital Frankfurt, Frankfurt am Main, Germany; 14 Division of Clinical Neurooncology, University Hospital Bonn, Bonn, Germany; 15 Division of Clinical Neurooncology, Department of Neurology, Center for Translational Neuro- and Behavioral Sciences (C-TNBS) and West German Cancer Center, Partner Site Essen, University Hospital Essen, German Cancer Consortium, University Duisburg-Essen, Essen, Germany; 16 Clinic and Polyclinic for Neurosurgery, University Hospital Carl Gustav Carus Dresden, Dresden, Germany; 17 Department of Neurosurgery, Charité Berlin, Berlin, Germany; 18 Department of Neurology, University Hospital Heidelberg, Heidelberg, Germany; 19 Department of Neuroradiology, Heidelberg University Hospital, Heidelberg, Germany; 20 Department of Neuropathology, University Hospital Heidelberg, Heidelberg, Germany; 21 Clinical Cooperation Unit Neuropathology, German Cancer Consortium (DKTK), German Cancer Research Center (DKFZ), Heidelberg, Germany; 22 National Center for Tumor Diseases (NCT), University Hospital Heidelberg, Heidelberg, Germany; 23 Helmholtz Institute for Translational Oncology (HI-TRON) Mainz, German Cancer Research Center, INF 280, D69120, Heidelberg, Germany

**Keywords:** Glioma, Central nervous system tumor, Diffuse midline glioma, T cell, Vaccine, Antigen, Immunotherapy, Atezolizumab

## Abstract

**Introduction:**

Diffuse midline gliomas (DMG) are universally lethal central nervous system tumors that carry almost unanimously the clonal driver mutation histone-3 K27M (H3K27M). The single amino acid substitution of lysine to methionine harbors a neoantigen that is presented in tumor tissue. The long peptide vaccine H3K27M-vac targeting this major histocompatibility complex class II (MHC class II)-restricted neoantigen induces mutation-specific immune responses that suppress the growth of H3K27M^+^ flank tumors in an MHC-humanized rodent model.

**Methods:**

INTERCEPT H3 is a non-controlled open label, single arm, multicenter national phase 1 trial to assess safety, tolerability and immunogenicity of H3K27M-vac in combination with standard radiotherapy and the immune checkpoint inhibitor atezolizumab (ATE). 15 adult patients with newly diagnosed K27M-mutant histone-3.1 (H3.1K27M) or histone-3.3 (H3.3K27M) DMG will be enrolled in this trial. The 27mer peptide vaccine H3K27M-vac will be administered concomitantly to standard radiotherapy (RT) followed by combinatorial treatment with the programmed death‐ligand 1 (PD-L1) targeting antibody ATE. The first three vaccines will be administered bi-weekly (q2w) followed by a dose at the beginning of recovery after RT and six-weekly administrations of doses 5 to 11 thereafter. In a safety lead-in, the first three patients (pts. 1–3) will be enrolled sequentially.

**Perspective:**

H3K27M-vac is a neoepitope targeting long peptide vaccine derived from the clonal driver mutation H3K27M in DMG. The INTERCEPT H3 trial aims at demonstrating (1) safety and (2) immunogenicity of repeated fixed dose vaccinations of H3K27M-vac administered with RT and ATE in adult patients with newly diagnosed H3K27M-mutant DMG.

**Trial registration:**

NCT04808245.

**Supplementary Information:**

The online version contains supplementary material available at 10.1186/s42466-023-00282-4.

## Introduction

Diffuse midline gliomas (DMG) are universally lethal central nervous system tumors that occur predominantly in children and young adults. The vast majority of these tumors are characterized by a clonal driver mutation in canonical (H3.1) or noncanonical (H3.3) histone-3 (H3K27M) that causes widespread epigenomic changes and deregulated gene expression [1]. Surgical treatment options are limited by the location, infiltrative growth, and aggressive phenotype of DMG rendering standard RT as the only therapeutic option with proven clinical benefit [2, 3]. Despite best medical treatment, prognosis remains poor with a median overall survival of less than one year, and a 2-year survival rate of less than 10% [4, 5].

Much like in other forms of malignant glioma [6, 7], immune checkpoint inhibition failed to yield survival benefits for patients with DMG [8] though several clinical trials evaluating the efficacy of PD-L1 blockade in DMG are currently recruiting patients (NCT02359565, NCT02793466, NCT03130959 and NCT01952769). This is likely due to the low tumor mutational burden rendering the tumor microenvironment immunologically ‘cold’ [9]. Therefore, novel immunotherapeutic strategies aim at overcoming these challenges by specifically targeting (neo)epitopes and tumor-associated cell surface proteins [10, 11]. Such approaches include disialoganglioside GD2 chimeric antigen receptor (CAR) T cell therapy [12] and peptide vaccinations against the clonal driver mutation H3K27M (Grassl et al. Nature Medicine, [13], Boschert et al. Science Advances, [14]). While a short H3.3K27M^26–35^ peptide vaccine has been shown to elicit CD8^+^ T cell responses against H3.3K27M in children with newly-diagnosed HLA-A*02:01^+^, H3.3K27M^+^ DMG [15], the recognition and killing of H3.3K27M mutant cells by these CD8^+^ T cell remains controversial [16].

Mounting evidence suggests a vital role of major histocompatibility complex (MHC) class II-restricted antigen presentation for effective cancer immunotherapy [17–19]. A long peptide vaccine, H3K27M-vac, that covers amino acids 14 to 40 of K27M-mutant histone-3 elicits predominantly CD4^+^ T cell-mediated immune responses in MHC-humanized mice. Vaccinated mice with H3K27M-expressing flank tumors displayed effective tumor control and post-vaccine-tumors were infiltrated with H3K27M-reactive T cells [20]. The administration of H3K27M-vac to eight adult patients with progressive DMG after radiation and alkylating chemotherapy on a compassionate use basis was well tolerated (Grassl et al. Nature Medicine, [13]). Furthermore, five of eight patients had H3K27M-reactive CD4^+^ T cells that expanded in peripheral blood upon vaccination and were also detectable in the CSF (Grassl et al. Nature Medicine, [13], Boschert et al. Science Advances, [14]).

## Methods

### Aim of the trial

INTERCEPT H3 assesses safety and immunogenicity of H3K27M-vac in combination with standard RT and with ATE in adult patients with newly diagnosed H3K27M-mutant DMG.

### Study description and study design

**Fig. 1:**
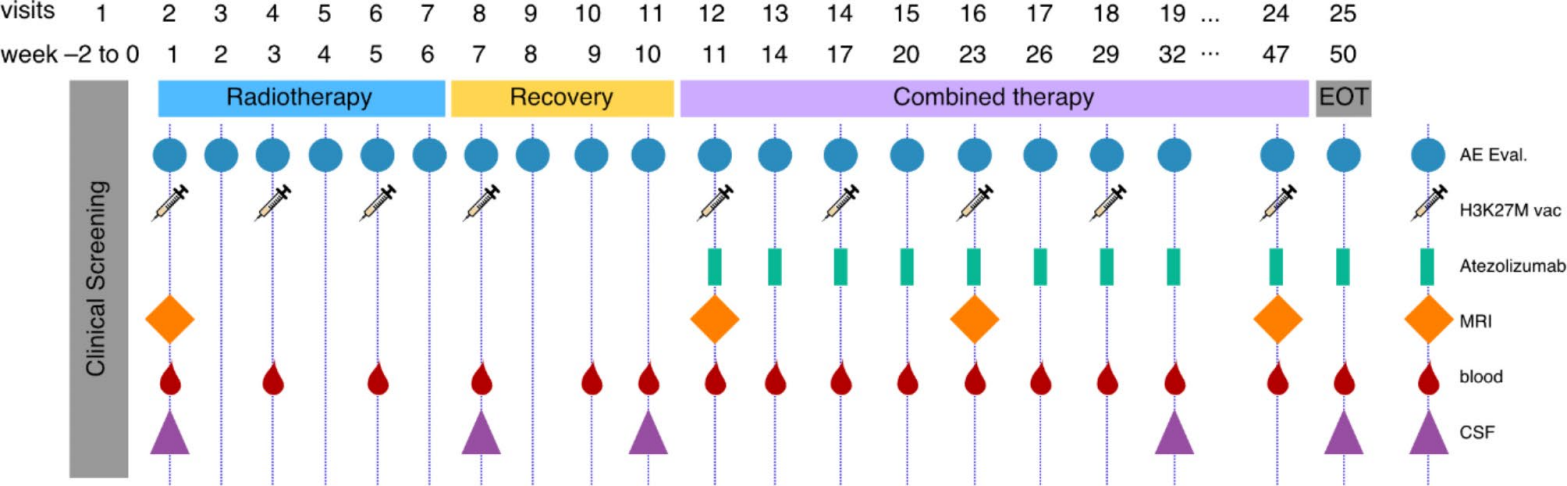
Intercept H3 treatment schedule H3K27M-vac will be administered to 15 patients with newly diagnosed H3K27M mutant DMG concomitant to standard radiotherapy and subsequently in combination with ATE. Longitudinal blood and CSF sampling will allow immunogenicity testing and three-monthly MRIs together with regular clinical evaluation will be used to determine clinical response

The non-controlled open label, single arm, multicenter national phase 1 trial will enroll 15 patients with newly diagnosed H3.1K27M- or H3.3K27M-mutant DMG, WHO grade 4. Patients must not have received previous treatment except for surgery. The trial treatment consists of subcutaneous injections of the long peptide vaccine H3K27M-vac in addition to standard RT. Thereafter, patients will receive a combination of the human anti-PD-L1 antibody ATE and H3K27M-vac. The primary treatment phase comprises 50 weeks (last administration in week 47). Patients will receive eleven doses of H3K27M-vac. The first four vaccines will be administered bi-weekly (q2w) concomitant to and after RT. After a four-weeks recovery period, seven vaccinations are given in six-week intervals (q6w) in combination with 14 doses of ATE administered every three weeks (q3w) (Fig. 1). Safety, tolerability and disease activity will be assessed by routine blood analyses and clinical evaluation every two to six weeks depending on treatment phase and MRI every three months. For primary immunogenicity endpoint and exploratory objectives, longitudinal blood and cerebrospinal fluid (CSF) sampling will be performed (Fig. 1). In a safety lead-in, the first three patients will be enrolled sequentially. Eight German trial sites within the German Cancer Consortium (DKTK) and the Neurooncology Working Group of the German Cancer Society (NOA) will offer study treatment.

### Arms and intervention

Following unequivocal diagnosis of diffuse midline glioma with H3K27M mutation proven by immunohistochemistry or H3 DNA sequencing, patients will be screened for eligibility upon written informed consent. After study enrollment, patients will be assigned to the single treatment arm with combinatorial treatment of H3K27M-vac, ATE and RT. H3K27M-vac consists of 300 µg of a 27-mer H3K27M (p14-40) peptide emulsified in Montanide (ISA50) to a total volume of 1 ml. The vaccine is injected subcutaneously in the thigh or abdomen followed by topical administration of imiquimod, a toll-like receptor 7/8 agonist (5%, Aldara®) [21]. A total of 250 mg of Aldara® cream will be applied to an area of 5 × 5 cm around the injection site 15 min after vaccination and left on the skin for approximately 8 h. 24 h after the vaccination a second sachet of Aldara® will be applied by the patient as instructed above and left on the skin for approximately 8 h. ATE (Tecentriq®, 1200 mg) will be administered intravenously over 60 min every 3 weeks starting 4 weeks after RT. If the first infusion is well tolerated, subsequent infusions will be delivered over 30 min. Disease course will be determined according to immunotherapy response assessment in neuro-oncology (iRANO) criteria.

### Outcome measures

Primary outcome measures will be safety and immunogenicity of H3K27M-vac in combination with RT and ATE assessed at the date of study termination.

For safety assessment, patients will be medically reviewed at each visit including recording of concomitant medications and adverse events (AE). All AE will be graded according to National Cancer Institute Common Terminology Criteria for Adverse Events (CTCAE) version 5.0. The primary safety endpoint is the Regime Limiting Toxicity (RLT) until end of the recovery phase in week 10. RLT is defined as any of the following if considered related to the administration of H3K27M-vac: (1) any injection site reaction of CTCAE grade 4; (2) any injection site reaction ≥ CTCAE grade 3 that persists after four weeks; (3) any other hypersensitivity, anaphylaxis or local allergic reaction ≥ CTCAE grade 3; (4) brain edema of CTCAE grade 4; (5) autoimmunity ≥ CTCAE grade 3; (7) ≥ CTCAE grade 3 toxicity to organs other than the bone marrow, but excluding the following: transient (≤ 6 h) CTCAE grade 3 flu-like symptoms or fever, which are controlled with medical management, grade 3 nausea, grade 3 or 4 vomiting in patients who have not received optimal treatment with anti-emetics, grade 3 or 4 diarrhea in patients who have not received optimal treatment with anti-diarrheas, transient (≤ 24 h) grade 3 fatigue, local reactions, headache, nausea, emesis that resolves to grade ≤ 1, single laboratory values out of normal range (excluding grade ≥ 3 liver function test increase) that resolve to grade ≤ 1 within 7 days with adequate medical management, tumor flare phenomenon defined as local pain, irritation, or rash localized at sites of known or suspected tumor; and (8) death (including death due to disease progression). Dose de-escalation of the trial agents is not allowed, but administration may be skipped because of AE. Secondary safety endpoints include the frequency and severity of AE, occurrence of serious AE, their suspected relationship to the study medication, as well as changes in laboratory parameters.

**Fig. 2:**
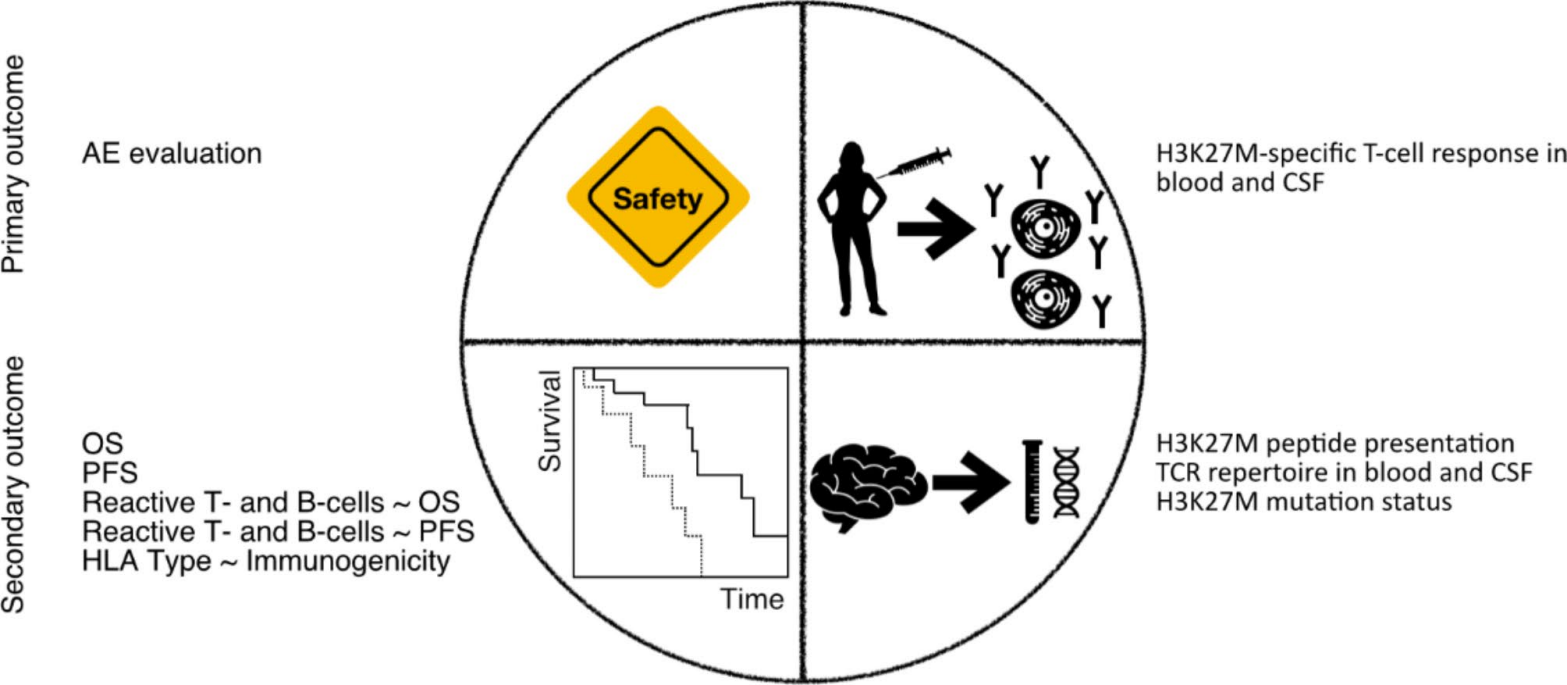
Summary of outcome measures of Intercept H3 Primary outcome measures comprise of safety and immunogenicity. Secondary outcomes are the efficacy measures OS, PFS, their relation to H3K27M-specific T- and B- cells in blood and CSF as well as the relation between HLA type and immunogenicity. Translational research goals include the analysis of H3K27M presentation and mutation status in recurrent tumors as well as the T cell repertoire in recurrent tumors if reoperation or biopsy is clinically indicated and tissue is available

The primary immunogenicity endpoint is the presence of an H3K27M-specific T cell response at any time point during the trial. H3K27M-specific T cell responses are measured on Peripheral Blood Mononuclear Cells (PBMC) using IFN-gamma Enzyme-linked-immuno-Spot (ELISpot) assay (Fig. 2).

Secondary outcome measures of the study include (1) progression free survival (PFS), (2) overall response rate (ORR), defined as the proportion of patients showing complete response, partial response or stable disease at end of study compared to MRI at visit 1, (3) association between immunogenicity and ORR, (4) association between immunogenicity and PFS.

To analyze biological activity in more detail, H3K27M-reactive T cell subtypes and H3K27M-reactive antibody subtypes in peripheral blood and CSF will be characterized. Immunogenicity will be related to the HLA-type and to the presence of H3K27M DNA in the peripheral circulation, furthermore clinical outcome to the presence of H3K27M-reactive T cells within the CSF as well as to the presence of H3K27M DNA in the peripheral circulation. In addition, H3K27M-specific antibody and B cell responses will be assessed at any time point during the trial measured by Enzyme-linked Immunosorbent Assay (ELISA) and patient-specific H3K27M-reactive T cell receptors will be characterized, benchmarked and functionally validated. Finally, functionality of vaccine-induced T cell receptors will be correlated with clinical outcome parameters.

Translational research objectives of the trial comprise the assessment of H3K27M immunoreactivity, H3K27M peptide presentation and H3K27M-specific TCR repertoire and phenotype in recurrent tumors, if re-resection or biopsy is clinically indicated and tissue is available. Furthermore, H3K27M mutation status in recurrent tumors and H3K27M-reactive T cell receptors in recurrent tumors will be analyzed in that case.

### Eligibility criteria

The main patient inclusion criteria are patient age ≥ 18 years, histologically confirmed diagnosis of an H3.1K27M or H3.3K27M-mutated diffuse midline glioma WHO grade 4 (with or without measurable residual tumor after tumor resection or biopsy after primary diagnosis), no previous treatment except for surgery, availability of tumor tissue for translational analyses (FFPE bulk tissue or biopsy), Patients are scheduled to receive RT, Patients should be immunocompetent (steroid levels must not exceed 2 mg/day dexamethasone), Patients should have a Karnofsky Performance Status of at least 60.

### Contacts

Sponsor: German Cancer Research Center, Im Neuenheimer Feld 280, 69,120 Heidelberg.

Investigators: Michael Platten, Neurology Clinic, Medical Faculty Mannheim, University Heidelberg; Katharina Sahm, Neurology Clinic, Medical Faculty Mannheim, University Heidelberg.

## Perspectives

The lysine to methionine substitution constitutes an attractive therapeutic target, because it is a clonal driver mutation. Preliminary data suggests that the H3K27M neoepitope can be safely targeted with H3K27M-vac following standard-of-care (SOC) treatment and that the vaccine induces peripheral mutation-specific T cell responses that translate into CNS-specific immunity (Grassl et al. Nature Medicine, [13]). Similar to the NOA-16 trial exploring safety and immunogenicity of IDH1-vac, a long peptide vaccine targeting the clonal driver mutation IDH1R132H [21, 22], INTERCEPT-H3 trial aims at investigating safety and immunogenicity of H3K27M-vac integrated into SOC in patients with newly diagnosed DMG. Based on preclinical observations that RT facilitates the accumulation and activity of tumor-infiltrating T cells [23], the vaccine will be initiated during RT and not following RT as done in NOA-16. As NOA-21, a neoadjuvant window-of-opportunity trial in patients with recurrent IDH1-mutant gliomas [24], INTERCEPT-H3 integrates an immune checkpoint inhibitor to amplify neoepitope-specific T cell responses. Exploratory HLA and pre-treatment tissue analyses will compare presentation of H3K27M neoepitope in the tumor microenvironment, immune cell infiltration and assess potential predictive biomarkers. Moreover, T cells isolated from peripheral blood and CSF at different timepoints will uncover the temporal dynamics of H3K27M-specific immune responses as well as crossing of T cell clones across the blood brain barrier. To our knowledge this is the first clinical trial aiming at eliciting CD4^+^ T cell dominated immune responses against the clonal driver mutation H3K27M in diffuse midline glioma and the first trial to investigate the combined treatment with H3K27M-specific peptide vaccine, RT and anti-PD-L1 therapy in DMG.

### Electronic supplementary material

Below is the link to the electronic supplementary material.

Supplementary Material 1

## Data Availability

Not applicable.

## Abbreviations

AE: Adverse events
ATE: Atezolizumab
CAR: Chimeric antigen receptor
CTCAE: Common Terminology Criteria for Adverse Events
CSF: Cerebrospinal fluid
DMG: Diffuse midline glioma
H3K27M: K27M-mutant histone-3
MHC: Major histocompatibility complex
MRI: Magnetic resonance imaging
HLA: Human leukocyte antigen
ORR: Overall response rate
PBMC: Peripheral blood mononuclear cells
PD-L1: Programmed death-ligand 1
RANO: Response assessment in neurooncology
RLT: Regimen-limiting toxicity
RT: Radiotherapy
SOC: Standard of care
TCR: T cell receptor
WHO: World Health Organization
