# Supplementary material for: INTERCEPT H3: a multicenter phase I peptide vaccine trial for the treatment of H3-mutated diffuse midline gliomas
Source: Neurol Res Pract. 2023 Oct 19;5:55. doi: 10.1186/s42466-023-00282-4 (PMC10585906; doi:10.1186/s42466-023-00282-4)
Supplement: Supplementary file 1 — Supplementary Material 1 [file 42466_2023_282_MOESM1_ESM.docx]

**Clinical Cooperation Unit Neuroimmunology and Brain Tumor Immunology**

D170

Head:

Prof. Dr. med. Michael Platten

Im Neuenheimer Feld 280

69120 Heidelberg

Germany

Phone	+49 6221 42-0000

Telefax	+49 6221 42-0000

Katharina.Sahm@dkfz.de

[www.dkfz.de](http://www.dkfz.de)

Heidelberg, 06/09/2023

**INTERCEPT H3 clinical trial protocol**

German Cancer Research Center | M123 | PO Box 101949 | 69009 Heidelberg | Germany

Neurological Research and Practice

Werner Hacke, MD

Editor-in-chief

Dear Prof. Hacke,

herewith we are submitting our manuscript entitled

*“INTERCEPT H3: A multicenter phase I peptide vaccine trial for the treatment of H3-mutated diffuse midline gliomas”*

for consideration to be published in Neurological Research and Practice.

In this manuscript, we describe the scientific background and design of our multicenter phase I clinical trial assessing safety and immunogenicity of a neoepitope-specific long peptide vaccine in combination with radiotherapy and Atezolizumab for the treatment of adult patients with newly diagnosed H3K27M-mutant diffuse midline gliomas.

Our manuscript presents the trial schedule in great detail and discusses the relevance of immunotherapeutic approaches targeting recurrent neoepitopes in neurooncology.

We thus think that this manuscript may be of interest to the readers of Neurological Research and Practice.

Thank you very much for your consideration.

Yours sincerely,

Michael Platten
